# Extremal states and coupling properties in electroelasticity

**DOI:** 10.1098/rsta.2021.0330

**Published:** 2022-10-17

**Authors:** A. Menzel, C. Witt

**Affiliations:** ^1^ Institute of Mechanics, TU Dortmund, Leonhard-Euler-Strasse 5, Dortmund 44227, Germany; ^2^ Division of Solid Mechanics, Lund University, PO Box 118,Lund 221 00, Sweden

**Keywords:** Ogden material, dielectric elastomer, extremal energy states, harmonic decomposition, deviators

## Abstract

Electroelastic materials possess properties most attractive for the design of smart devices and systems such as actuators and sensors. Typical polymers show changes in shape under the action of an electric field, and vice versa, together with fast actuation times, high strain levels and low elastic moduli. This paper deals with an Ogden model inspired framework for large deformation electroelasticity which, as a special case, can also be reduced to the modelling of transversely isotropic elasticity. Extremal (local) states are elaborated based on a coaxiality analysis, i.e. extremal states of energy are considered at fixed deformation and changing direction of electric field, respectively, fixed electric field and changing principal directions of deformation. This analysis results in extremal states when stresses and strain commutate, respectively, dielectric displacements and electric field are aligned. In order to further elaborate electromechanical coupling properties, the sensitivity of stresses with respect to electric field is analysed. This sensitivity is represented by a third-order tensor which, in general, depends on deformation and electric field. To illustrate this third-order tensor, a decomposition into deviators is adopted. Related norms of these deviators, together with the electromechanical coupling contribution to the augmented energy, are investigated for different states under homogeneous deformation and changing electric field direction. The analysis is considered to contribute to a better understanding of electromechanical coupling properties and extremal states in large deformation electroelasticity and by that, as a long-term goal, may contribute to the improved design of related smart devices and systems.

This article is part of the theme issue ‘The Ogden model of rubber mechanics: Fifty years of impact on nonlinear elasticity’.

## 1. Introduction

Electroelasticity deals with the modelling and simulation of electromechanical coupling for reversible processes of solid continua. Electromechanical coupling in general features challenging phenomena such as changes in shape of a solid continuum under the action of an electric field, and vice versa, together with fast actuation. These electromechanical coupling properties in combination with high strain levels and low elastic moduli make such materials attractive for the design of smart devices such as actuators and sensors. Investigations on related electroactive polymer-based smart systems, not necessarily restricted to dissipation-free electroelasticity, include artificial muscles and grippers, [1–3], sensors, [4], tuneable lenses, [5], loudspeakers, [6] and three-dimensional printed actuator systems [7], to name but a few.

The modelling of electroelasticity is embedded into the framework of nonlinear continuum mechanics at large deformations and based on mechanical balance relations, such as balance of linear momentum, in combination with Maxwell’s equations which, for the problem at hand, are reduced to the basic case of Gauß’s law; see e.g. [8–11]. For an overview on the modelling of electroelasticity the reader is referred to Dorfmann & Ogden [12] and references cited therein. An assumption often made, and also adopted as this work proceeds, within the modelling of dielectric elastomers is considering the electroelastic energy to be significantly larger than self-interaction energy contributions—in other words, local energy relations are introduced and self-interaction contributions are neglected. For detailed background on theories including dependencies on the full state of polarization of the entire body through Maxwell’s equations, see e.g. [13–15] and references cited therein. Modelling approaches for electroelasticity intrinsically include contributions related to the purely elastic response, the electromechanical coupling behaviour and, possibly, solely electrical contributions. The (phenomenological) modelling of the purely elastic and isotropic response is well understood and the celebrated Ogden model, [16,17], enables maximum flexibility in view of matching related experimental data to model predictions in combination with computational efficiency. Concerning the electromechanical coupling contribution, however, comprehensive modelling approaches based on the theory of tensor functions are established, see e.g. [12], but typically reduced to energy contributions including a few basic combinations of the underlying invariants. A different approach, based on a so-called micro-sphere framework, which includes coupling of powers of electric field and stretch contributions, whereby the values of the powers are determined by a parameteridentification approach, is proposed in [18]. The electromechanical coupling properties are intrinsically related to deformation dependency of the electric permittivity as investigated in, e.g. [19–22]. Further experimental investigations on the electromechanical coupling response and rate dependent behaviour are discussed in, e.g. [23,24], whereas [25] also addresses temperature dependencies. Different modelling frameworks in combination with parameter identification approaches are proposed to model electro-viscoelasticity, see e.g. [26,27], and thermo-electro-viscoelasticity, see [28].

Common to models predicting the electromechanical response of polymers is their anisotropic response in the sense that conjugated stress and strain tensors do not commutate, or commutate only in very specific situations, respectively. Such states where conjugated stresses and strains commutate correspond to a related property between dielectric displacements and electric field on the one hand and are of interest for the design of related smart devices on the other. In order to investigate such extremal energy states, the coaxiality approach introduced in [29,30] can be adopted, see also [31].

Electromechanical coupling, which is the fundamental property of main importance for any electromechanical device, is directly reflected by the sensitivity of stresses with respect to electric field, and by the sensitivity of dielectric displacements with respect to deformation for the class of materials considered in this work. From a mechanics and modelling point of view, this sensitivity is represented by a third-order tensor. In the case of nonlinear electroelasticity, such third-order tensors are, in general, not constant but depend on deformation and electric field. In order to better understand electromechanical coupling properties by means of such third-order tensors, suitable illustrations are useful. While related visualization techniques are frequently used for (symmetric) second-order tensors and, partly, also for (symmetric) fourth-order tensors. Similar visualization tools for third-order tensors are, to the knowledge of the authors, not well established in the mechanics community. A typical basis for tensor visualization is a suitable tensor decomposition. Examples are spectral decompositions and harmonic, respectively, irreducible decompositions, cf. [32,33] and [34,35] including decompositions of higher-order tensors. The underlying deviators of irreducible decompositions can be represented by vectorial (Maxwell) multipoles, see [36]. Applications of multipole representations are discussed in detail in, e.g. [37] for third-order piezoelectric tensors and in, e.g. [38] with an emphasis on fourth-order elasticity tensors. Based on these multipole representations, advanced visualization approaches have been proposed, cf. [39,40].

The main goal of the present paper is to elaborate extremal (local) states of energy in the context of large deformation electroelasticity and to analyse electromechanical coupling based on the sensitivity of stresses with respect to electric field as represented by a third-order tensor. Discussing and better understanding such extremal states and electromechanical coupling properties is considered to be useful in view of the design of related smart devices such as electroelastic actuators and sensors. Along these lines, first basic field and modelling relations of electroelasticity at finite deformations are summarized in §2. Constitutive relations are derived from an augmented energy function in §3. First, the augmented energy function is introduced in general invariant-based form and then particularized to an Ogden model type formulation. As a special case, transversely isotropic elasticity is included for normalized electric field. With the fundamental modelling relations introduced, general relations of extremal (local) states of energy are investigated in §4. Moreover, electromechanical coupling properties shall be analysed by the third-order tensor obtained from the derivative of stresses with respect to electric field. For the purposes of illustration and interpretation, this third-order tensor is decomposed into deviators. Use of multipole representations, as mentioned above, is not the focus of this paper, so that the examples discussed in §5 place emphasis on the loading state dependent norm of the respective deviators only, together with the illustration of the electromechanical coupling contribution to the augmented energy. For the purposes of illustration, homogeneous states of deformation are considered in combination with changing orientations of electric field. Finally, the paper closes with a summary in §6.

## 2. Basic field and modelling relations

In the following, basic field and modelling relations for electroelasticity are briefly summarized. This includes essential balance relations in local form and the continuum mechanics background used. Standard notation is adopted in the sense that contraction operations are represented by one ⋅ and dyadic (tensor) products shall be denoted by ⊗, for example $\left[v_{1}⊗v_{2}]:[v_{3}⊗v_{4}]=[v_{1}\cdot v_{3}][v_{2}\cdot v_{4}\right]$ wherein $v_{1},\ldots ,v_{4}$ represent vectors. In addition to the standard dyadic product, the notations $[v_{1}⊗v_{2}]\;\bar{⊗}\;[v_{3}⊗v_{4}]=v_{1}⊗v_{3}⊗v_{2}⊗v_{4}$, $[v_{1}⊗v_{2}]\;\underline{⊗}\;[v_{3}⊗v_{4}]=v_{1}⊗v_{3}⊗v_{4}⊗v_{2}$ and $v_{1}⊗v_{2}\;\bar{⊗}\;v_{3}=v_{1}⊗v_{3}⊗v_{2}$, $v_{1}\;\bar{⊗}\;v_{2}⊗v_{3}=v_{2}⊗v_{1}⊗v_{3}$ are introduced. Moreover, transpositions are used in the form ${\left[v_{1}⊗v_{2}\right]}^{t}=v_{2}⊗v_{1}$, ${\left[v_{1}⊗v_{2}⊗v_{3}\right]}^{t}=v_{1}⊗v_{3}⊗v_{2}$, ${\left[v_{1}⊗v_{2}⊗v_{3}⊗v_{4}\right]}^{t}=v_{1}⊗v_{2}⊗v_{4}⊗v_{3}$, ${\left[v_{1}⊗v_{2}⊗v_{3}⊗v_{4}\right]}^{T}=v_{3}⊗v_{4}⊗v_{1}⊗v_{2}$ and ${\left[v_{1}⊗v_{2}⊗v_{3}\right]}^{T}=v_{2}⊗v_{3}⊗v_{1}$, ${\;}^{T}[v_{1}⊗v_{2}⊗v_{3}]=v_{3}⊗v_{1}⊗v_{2}$.

Concerning the kinematics of the solid continuum body B considered, placements in referential configuration of material points are denoted by $X\in B_{0}$, whereas related spatial placements at time t are introduced as $x=\varphi (X,t)\in B_{t}$. Moreover, the deformation gradient is denoted by $F=\nabla_{X}\varphi$, with J=det(F)>0 and $\mathrm{cof}(F)=JF^{-t}$. Use shall be made of the right Cauchy–Green tensor $C=F^{t}\cdot F$ as well as of the left Cauchy–Green, Finger tensor $b=F\cdot F^{t}$. It is remarked that a volumetric-isochoric multiplicative decomposition of the deformation gradient, i.e. $F=F^{\mathrm{vol}}\cdot F^{\mathrm{iso}}$ with $F^{\mathrm{vol}}=J^{1/3}I$ and I denoting the second-order identity tensor, results in $C^{\mathrm{iso}}=J^{-2/3}C$ and $b^{\mathrm{iso}}=J^{-2/3}b$. For further background on the kinematics of deformation in the context of nonlinear continuum mechanics, the reader is referred to, e.g. [9,41].

Conservation of mass is assumed and the essential balance equations considered are reduced to the Gauß’s law and the balance of linear momentum, both in stationary, respectively, quasi-static form. This yields the electric field irrotational so that the electric field can be introduced as the gradient of a scalar field, i.e. the electric potential. To be specific, the spatial electric field allows representation as $e=-\nabla_{x}\phi$ which yields its referential representation as $E=e\cdot F=-\nabla_{X}\phi$. Specifying Gauß’s law in local form for the particular case considered, i.e. neglecting free charges, yields the spatial and referential representations

2.1 $$\nabla_{x}\cdot d=0\;\text{and}\;\nabla_{X}\cdot D=0,$$

wherein d, respectively, D=d⋅cof(F) denote dielectric displacements. The universal relation between (spatial) dielectric displacements and electric field is provided by $d=\varepsilon_{0}e+\pi$, including the polarization π and the permittivity of free space $\varepsilon_{0}\approx 8.854\times 10^{-12}\;N\;V^{-2}$. With these relations in hand, electric volume (Kelvin) forces are considered which, in local form, read

2.2 $$f_{e}=\nabla_{x}e\cdot \pi =\nabla_{x}\cdot t_{e}\;\text{with}\;t_{e}=e⊗d-\frac{1}{2}\varepsilon_{0}[e\cdot e]I.$$

These electric volume forces contribute, in addition to mechanical volume forces, to the balance of linear momentum which, neglecting acceleration contributions, yields the spatial and referential local form representations

2.3 $$\nabla_{x}\cdot \sigma +\varrho_{t}f=0\;\text{and}\;\nabla_{X}\cdot P+\varrho_{0}f=0,$$

wherein $\varrho_{t}$ and $\varrho_{0}=J\varrho_{t}$ denote the spatial and referential mass density, respectively. Mechanical volume forces are represented by f, in other words use is made of the divergence based representation of electrical volume force contribution in equation (2.2), so that the Cauchy type stresses may additionally be decomposed into $\sigma =t+t_{e}=P\cdot \mathrm{cof}(F^{-1})$. It is remarked that this decomposition of stresses is arbitrary and a clear distinction between mechanical and electrostatic contributions is, in general, impossible.

As this work proceeds, the framework of introducing an augmented energy function $\Omega_{0}(C,E)$ shall be adopted, cf. [42,43]. To be specific, the free space contribution is included via $\Omega_{0}(C,E)=\Psi_{0}(C,E)+\Omega_{0}^{\text{fre}}(C,E)$ with $\Omega_{0}^{\text{fre}}(C,E)=-\frac{1}{2}\varepsilon_{0}JE\cdot C^{-1}\cdot E$. Following the lines of continuum thermodynamics, Piola-type stresses and referential dielectric displacements take the representations

2.4 $$P=\frac{\partial \Omega_{0}}{\partial F}\;\text{and}\;D=-\frac{\partial \Omega_{0}}{\partial E}.$$

In the following, focus shall additionally be placed on the Piola–Kirchhoff type stresses $S=F^{-1}\cdot P=2\partial \Omega_{0}/\partial C=S^{t}$.

Material properties are reflected by sensitivity-type (material) tensors, i.e. sensitivities related to changes of stresses with deformation (elasticity tensor), of dielectric displacements with electric field (electric permittivity) and of stresses with electric field, respectively, of dielectric displacements with deformation (electromechanical coupling tensor). These tensors can be introduced as

2.5 $$\text{A}=\frac{\partial P}{\partial F}=2F\cdot \frac{\partial S}{\partial C}:[F^{t}\bar{⊗}I]+I\bar{⊗}S,\;\text{B}=\frac{\partial P}{\partial E}=F\cdot \frac{\partial S}{\partial E}\;\text{and}\;K=\frac{\partial D}{\partial E}.$$

Since (anisotropic) material properties are typically analysed with respect to the referential configuration, the referential material tensors

2.6 $$\text{E}=2\frac{\partial S}{\partial C}\;\text{and}\;\text{H}=\frac{\partial S}{\partial E}=-2[\frac{\partial D}{\partial C}{]}^{T}$$

as well as K are of particular interest for the analysis as this work proceeds. It is remarked that these material tensors can also be referred to the spatial configuration, respectively, sensitivities of σ and d with respect to deformation and electric field, i.e.

2.7 $$\text{e}=J^{-1}[F\bar{⊗}F]:\text{E}:[F^{t}\bar{⊗}F^{t}],\;\text{h}=J^{-1}[F\bar{⊗}F]:\text{H}\cdot F^{t}\;\text{and}\;k=J^{-1}\;F\cdot K\cdot F^{t}.$$

## 3. An Ogden model based electroelasticity formulation

The previously introduced augmented energy function $\Omega_{0}(C,E)$ can be specified as scalar-valued isotropic tensor function formulated in, e.g. basic invariants, namely

3.1 $$I_{1}=C:I,\;I_{2}=C^{2}:I,\;I_{3}=C^{3}:I$$

and

3.2 $$I_{4}=E\cdot E,\;I_{5}=E\cdot C\cdot E,\;I_{6}=E\cdot C^{2}\cdot E,$$

so that $\Omega_{0}(C,E)={\bar{\Omega }}_{0}(I_{1},\ldots ,I_{6})$. Alternative useful invariants, related to equations (3.1) and (3.2) via the Cayley–Hamilton theorem, are

3.3 $$J_{2}=\mathrm{cof}(C):I,\;J_{3}=\det(C)=J^{2}\;\mathrm{and}\;J_{6}=E\cdot C^{-1}\cdot E,$$

with $J_{6}=J_{3}^{-1}[I_{6}-J_{1}I_{5}+J_{2}I_{4}]$ and, in addition, $J_{1}=I_{1}$. In view of a presentation in terms of principal stretches and a presentation in the spirit of the celebrated Ogden model, [16,17], a spectral decomposition of the right Cauchy–Green tensor is used in the form

3.4 $$C=\sum_{i=1}^{3}\lambda_{i}^{2}N_{i}⊗N_{i}\;\text{with}\;\lambda_{i}>0\;\text{and}\;N_{i}\cdot N_{j}=\delta_{ij},$$

wherein $\delta_{ij}$ denotes the Kronecker delta. Furthermore, one may also refer the representation of the referential electric field to the principal directions $N_{i}$ of the right Cauchy–Green tensor, i.e.

3.5 $$E=\sum_{i=1}^{3}E_{i}N_{i}.$$

This enables the presentation of the basic invariants in equations (3.1) and (3.2) via principal stretches and related electric field coefficients as

3.6 $$I_{1}=\lambda_{1}^{2}+\lambda_{2}^{2}+\lambda_{3}^{2},\;I_{2}=\lambda_{1}^{4}+\lambda_{2}^{4}+\lambda_{3}^{4},\;I_{3}=\lambda_{1}^{6}+\lambda_{2}^{6}+\lambda_{3}^{6}$$

and

3.7 $$I_{4}=E_{1}^{2}+E_{2}^{2}+E_{3}^{2},\;I_{5}=E_{1}^{2}\lambda_{1}^{2}+E_{2}^{2}\lambda_{2}^{2}+E_{3}^{2}\lambda_{3}^{2},\;I_{6}=E_{1}^{2}\lambda_{1}^{4}+E_{2}^{2}\lambda_{2}^{4}+E_{3}^{2}\lambda_{3}^{4},$$

whereas the invariants introduced in equation (3.3) result in

3.8 $$J_{2}=\lambda_{1}^{2}\lambda_{2}^{2}+\lambda_{2}^{2}\lambda_{3}^{2}+\lambda_{3}^{2}\lambda_{1}^{2},\;J_{3}=\lambda_{1}^{2}\lambda_{2}^{2}\lambda_{3}^{2}\;\mathrm{and}\;J_{6}=E_{1}^{2}\lambda_{1}^{-2}+E_{2}^{2}\lambda_{2}^{-2}+E_{3}^{2}\lambda_{3}^{-2}.$$

As this work proceeds, an additive decomposition of the augmented energy function into a purely elastic and an electromechanically coupled contribution together with the free space part is adopted, namely

3.9 $$\Omega_{0}(C,E)=\Psi_{0}^{\mathrm{ela}}(C)+\Psi_{0}^{\mathrm{elm}}(C,E)+\Omega_{0}^{\mathrm{fre}}(C,E)\;\text{with}\;\Omega_{0}^{\mathrm{fre}}(C,E)=-\frac{1}{2}\varepsilon_{0}JJ_{6}.$$

Although the different energy contributions cannot clearly be separated in experiments, such additive split of the energy function is common in the literature, see e.g. [28,43]. Moreover, an additional purely electric contribution shall be neglected as this work proceeds. It is remarked that the particularization of $\Psi_{0}^{\mathrm{ela}}(C)$ should guarantee vanishing elastic initial stress contributions and $\Psi_{0}^{\mathrm{elm}}(C,E)$ should result in vanishing electromechanical stress contributions for vanishing electric field, as is also guaranteed by the particularization of $\Omega_{0}^{\mathrm{fre}}(C,E)$ in equation (3.9). In view of a representation based on invariants, the general form of the elastic contribution is well-established, i.e.

3.10 $${\hat{\Psi }}_{0}^{\mathrm{ela}}(J_{1},J_{2},J_{3})=\sum_{p,q,r=0}^{\infty }a_{pqr}{\left[J_{1}-3\right]}^{p}{\left[J_{2}-3\right]}^{q}{\left[J_{3}-1\right]}^{r}$$

with constants $a_{pqr}$ typically constrained in order to guarantee, e.g. a stress-free initial state, cf. [44]. The electromechanical coupling part, however, is often kept comparatively simple rather than introducing more general representations. Particular extended formats including, e.g. deformation dependent permittivity are commonly directly specified; see also remark 3.1. An approach which multiplicatively combines powers of invariants quadratic in the referential electric field combined with purely deformation dependent functions $f_{4,5,6}(C)$, ${\hat{f}}_{4,5,6}(J_{1},J_{2},J_{3})$, may formally be introduced as

3.11 $${\hat{\Psi }}_{0}^{\mathrm{elm}}(J_{1},J_{2},J_{3},I_{4},I_{5},I_{6})=\sum_{s,u,v=0}^{\infty }b_{suv}\;{\hat{f}}_{4}\;I_{4}^{s}\;{\hat{f}}_{5}\;I_{5}^{u}\;{\hat{f}}_{6}\;I_{6}^{v}.$$

It is reasonable that $\Psi_{0}^{\mathrm{elm}}(C,E)$ does not generate stress contributions without coupling to the electric field, since these contributions are included in $\Psi_{0}^{\mathrm{ela}}(C)$. In this regard, $b_{000}=0$ is an appropriate choice. Moreover, the derivatives of $I_{4}$, $I_{5}$ and $I_{6}$ with respect to E contribute to the referential dielectric displacements D. At undeformed state, i.e. C=I, these derivatives reduce to 2E. One may assume that $D=-\partial \Omega_{0}/\partial E$ and E should possess identical direction for C=I, see also remark 3.1, which further constrains the parameters $b_{suv}$ together with the reasonable choice $f_{4,5,6}(C)>0$.

In order to further specify the respective energy contributions, an additive decomposition into a volumetric and an isochoric deformation related elastic energy contribution is adopted, i.e.

3.12 $$\begin{array}{rl} & \Psi_{0}^{\mathrm{ela}}(C)=\Psi_{0}^{\mathrm{vol}}(J)+\Psi_{0}^{\mathrm{iso}}(C^{\mathrm{iso}})\;\text{with} \end{array}$$

3.13 $$\begin{array}{rl} & C^{\mathrm{iso}}=\sum_{i=1}^{3}{\left[\lambda_{i}^{\mathrm{iso}}\right]}^{2}N_{i}⊗N_{i}\;\text{and}\;\lambda_{i}^{\mathrm{iso}}=J^{-(1/3)}\lambda_{i}. \end{array}$$

Based on this, the isochoric elastic energy contribution $\Psi_{0}^{\mathrm{iso}}(C^{\mathrm{iso}})$ can be particularized in the form of the celebrated Ogden model, namely

3.14 $${\tilde{\Psi }}_{0}^{\mathrm{iso}}(\lambda_{1}^{\mathrm{iso}},\lambda_{2}^{\mathrm{iso}},\lambda_{3}^{\mathrm{iso}})=\sum_{p=1}^{N}\frac{\mu_{p}}{\alpha_{p}}[{\left[\lambda_{1}^{\mathrm{iso}}\right]}^{\alpha_{p}}+{\left[\lambda_{2}^{\mathrm{iso}}\right]}^{\alpha_{p}}+{\left[\lambda_{3}^{\mathrm{iso}}\right]}^{\alpha_{p}}-3],$$

with material parameters $\mu_{p}$ and $\alpha_{p}$, cf. [41]. Motivated by equation (3.14), including the basic invariants of $C^{\mathrm{iso}}$, i.e. $I_{i}^{\mathrm{iso}}={\left[C^{\mathrm{iso}}\right]}^{i}:I$, for particular choices of α∈R, namely $\alpha_{p}=2,4,6$, an analogous specification shall be chosen for the electromechanical coupling contribution $\Psi^{\mathrm{elm}}(C,E)$ which is introduced to depend on C instead of $C^{\mathrm{iso}}$, cf. [45] with a focus on anisotropic elasticity. In case it is assumed that the respective contributions remain quadratic in the electric field, a possible assumption is

3.15 $${\tilde{\Psi }}_{0}^{\mathrm{elm}}(\lambda_{1},\lambda_{2},\lambda_{3},E_{1},E_{2},E_{3})=\sum_{u=1}^{M}\tilde{f}(\lambda_{1},\lambda_{2},\lambda_{3})\frac{\nu_{s}}{\beta_{s}}[E_{1}^{2}\lambda_{1}^{\beta_{s}}+E_{2}^{2}\lambda_{2}^{\beta_{s}}+E_{3}^{2}\lambda_{3}^{\beta_{s}}]$$

with material parameters $\nu_{s}$ and $\beta_{s}$, so that $I_{4,5,6}$ and $J_{6}$ are included for particular choices of $\beta_{s}\in R$, namely $\beta_{s}=0,2,4,-2$. Moreover, the electric field E has been chosen to enter the electromechanical energy contribution in quadratic form which could be extended to, e.g., higher powers so that the dielectric displacements D remain of odd order in E.

**Remark 3.1.**

A particular choice for the electomechanical coupling part is

3.16 $$\Psi_{0}^{\mathrm{elm}}=[\varepsilon_{r}-1]\Omega_{0}^{\mathrm{fre}}\;\text{such that}\;\Psi_{0}^{\mathrm{elm}}(C,E)+\Omega_{0}^{\mathrm{fre}}(C,E)=-\frac{1}{2}\varepsilon_{0}\varepsilon_{r}JJ_{6}.$$

This results in $D=\varepsilon_{0}\varepsilon_{r}JC^{-1}\cdot E$ and $d=\varepsilon_{0}\varepsilon_{r}e$, respectively, and yields (spatially) linear dielectric response for $\varepsilon_{r}=\mathrm{const}$. For such a linear dielectric case, the related contribution to the Piola–Kirchhoff type stress contribution reads $S^{\mathrm{elm}}+S^{\mathrm{fre}}=\varepsilon_{0}\varepsilon_{r}J[C^{-1}\cdot E⊗E\cdot C^{-1}-\frac{1}{2}J_{6}C^{-1}]$ and the corresponding Cauchy type stress contribution follows as $\sigma^{\mathrm{elm}}+\sigma^{\mathrm{fre}}=\varepsilon_{0}\varepsilon_{r}[e⊗e-\frac{1}{2}[e\cdot e]I]$ with $J_{6}=e\cdot e$. It is remarked that $\sigma^{\mathrm{elm}}+\sigma^{\mathrm{fre}}\neq t_{e}$, cf. equation (2.2), but a (Korteweg–Helmholtz) force in the form $\nabla_{x}\cdot [\sigma^{\mathrm{elm}}+\sigma^{\mathrm{fre}}]$ may be introduced as an alternative to the (Kelvin) force $f_{e}$.

More advanced specifications of the electromechanical coupling contribution reflect deformation dependent permittivity, i.e. $\varepsilon_{r}(C)$ which, in the present context, still yields the spatial dielectric displacements d possessing the same direction as the spatial electric field e. An example is $\varepsilon_{r}={\bar{\varepsilon }}_{r}[1+b[\lambda_{1}^{\mathrm{iso}}+\lambda_{2}^{\mathrm{iso}}+\lambda_{3}^{\mathrm{iso}}]-3b]$ with constants b and ${\bar{\varepsilon }}_{r}$, see e.g. [46]. It is remarked that $\varepsilon_{r}(C)$ results in additional stress contributions.

**Remark 3.2.**

The electromechanical modelling framework discussed can be applied, or rather reduced to transversely isotropic elasticity. Conceptually speaking, this includes the constraint $I_{4}=1$ so that E⊗E represents a classic structural tensor. Specifications of (locally) extremal states as well as particularizations of, e.g. stress tensors can be derived as special cases of the representations discussed for the electromechanically coupled case as this work proceeds.

## 4. Extremal local states of energy

As established for finite elasticity, locally extremal states of energy correspond to commutating stresses and (related) deformation tensors such as the Piola–Kirchhoff stress tensor S and the right Cauchy–Green tensor C, i.e. S⋅C=C⋅S, see e.g. [29–31]. By analogy with transversely isotropic elasticity, this can be directly transferred to electroelastic behaviour, which shall be analysed in the following. Moreover, such particular states are also reflected by symmetry properties of material tensors such as E, H and K. In the following, particular emphasis is placed on H since this third-order tensor directly reflects electromechanical coupling properties, in other words the sensitivity of stresses with respect to electric field and of dielectric displacements with respect to deformation, respectively.

### (a) Coaxiality of electric field and dielectric displacements

The basic idea to analyse locally extremal states of energy for the electromechanically coupled problem at hand consists in considering fixed states of deformation represented by C and identifying orientations of the electric field E that result in locally extremal states of energy. Alternatively, E could be fixed and C rotated, cf. remark 4.1. In this context, let Q be an orthogonal second-order tensor, i.e. $Q^{t}=Q^{-1}$, which shall be restricted to represent a rotation so that det(Q)=1. Such rotation tensor allows representation as Q=exp⁡(V), whereby the second-order tensor $V=-V^{t}$ is skewsymmetric. Furthermore, the directional derivative of $Q_{\eta }=\exp(\eta W)\cdot Q$ results in $\left(\frac{d}{d}\eta \right)Q_{\eta }{|}_{\eta =0}=W\cdot Q$ with the direction, and tangent, $W=-W^{t}$.

In view of notation, let the rotated electric field be represented by $E^{*}=Q\cdot E$. For C and E fixed, locally extremal states of energy correspond to

4.1 $$\frac{\partial \Omega_{0}(C,E^{*})}{\partial Q}{|}_{C,E}:[W_{I}\cdot Q]=0\;\forall \;W_{I}=-W_{I}^{t}.$$

Specification of equation (4.1) results in

4.2 $$[\frac{\partial \Omega_{0}(C,E^{*})}{\partial E^{*}}\cdot \frac{\partial E^{*}}{\partial Q}\cdot Q^{t}]:W_{I}=-[D^{*}⊗E^{*}]:W_{I}=0\;\forall \;W_{I}=-W_{I}^{t}$$

with $D^{*}=D(C,E^{*})$. Equation (4.2) is satisfied if $\left[D^{*}⊗E^{*}\right]$ is symmetric, in other words if $D^{*}$ and $E^{*}$ are colinear and coaxial, respectively.

In order to further specify the dielectric displacements $D^{*}=-\partial \Omega_{0}(C,E^{*})/\partial E^{*}$, use of equations (2.4), (3.1) and (3.2) is made which results in

4.3 $$-D^{*}=\sum_{i=1}^{6}\frac{\partial {\bar{\Omega }}_{0}(I_{1}^{*},\ldots ,I_{6}^{*})}{\partial I_{i}^{*}}\frac{\partial I_{i}^{*}}{\partial E^{*}}=2[{\bar{\Omega }}_{0,4}^{*}E^{*}+{\bar{\Omega }}_{0,5}^{*}C\cdot E^{*}+{\bar{\Omega }}_{0,6}^{*}C^{2}\cdot E^{*}]$$

with $I_{i}^{*}=I_{i}(C,E^{*})$ and ${\bar{\Omega }}_{0,i}^{*}=\partial {\bar{\Omega }}_{0}(I_{1}^{*},\ldots ,I_{6}^{*})/\partial I_{i}^{*}$. It is remarked that, in general, $D^{*}\neq Q\cdot D$.

Equation (4.3) clearly indicates that $D^{*}$ and $E^{*}$ are coaxial and $\left[D^{*}⊗E^{*}\right]$ is symmetric, in case $E^{*}$ is aligned with a principal direction $N_{i}$ of C. In such scenarios two of the coefficients $E_{i}^{*}$ of the electric field, as included in equations (3.7), (3.8) and (3.15), vanish identically. Moreover, for identical stretch values, i.e. $\lambda_{1}=\lambda_{2}=\lambda_{3}$, any direction of $E^{*}$ results in coaxiality of electric field and dielectric displacements. Similarly, two identical stretch values, say $\lambda_{2}=\lambda_{3}$, yield the electric field aligned with dielectric displacements for any $E^{*}$ within the plane perpendicular to the principal direction related to $\lambda_{1}$. Furthermore, $E^{*}$ aligned with a principal direction $N_{i}$ of C results in coaxiality of $S^{*}$ and C so that $S^{*}\cdot C=C\cdot S^{*}$ as, by analogy with equation (4.3), can be concluded from

4.4 $$\begin{array}{rl} S^{*} & \;=2\sum_{i=1}^{6}\frac{\partial {\bar{\Omega }}_{0}(I_{1}^{*},\ldots ,I_{6}^{*})}{\partial I_{i}^{*}}\frac{\partial I_{i}^{*}}{\partial C}\\ & \;=2[{\bar{\Omega }}_{0,1}^{*}I+2{\bar{\Omega }}_{0,2}^{*}C+3{\bar{\Omega }}_{0,3}^{*}C^{2}+{\bar{\Omega }}_{0,5}^{*}E^{*}⊗E^{*}+{\bar{\Omega }}_{0,6}^{*}[C\cdot E^{*}⊗E^{*}+E^{*}⊗E^{*}\cdot C]], \end{array}$$

with, in general, $S^{*}\neq Q\cdot S\cdot Q^{t}$.

One may further compare energy levels related to extremal states by simple evaluation of energy functions at the respective extremal states. More rigorously, a fourth-order Hessian-type tensor can be analysed related to a second directional derivative by analogy with equation (4.1) at an extremal state. To be specific, such a second directional derivative results in

4.5 $$-W_{I}:[\frac{\partial [D^{*}⊗E^{*}]}{\partial Q}\cdot Q^{t}]:W_{II}=-W_{I}:{\text{T}}^{*}:W_{II}$$

with

4.6 $${\text{T}}^{*}=K^{*}\;\bar{⊗}\;E^{*}⊗E^{*}+D^{*}⊗I⊗E^{*}$$

and $K^{*}=\partial D^{*}/\partial E^{*}$ as well as $W_{II}=-W_{II}^{t}$.

**Remark 4.1.**

Instead of analysing extremal states of energy by rotating the electric field at fixed deformation tensor, one may also rotate the right Cauchy–Green tensor, i.e. $C^{⋆}=Q\cdot C\cdot Q^{t}$, and fix C and E. By analogy with equation (4.1), considering $\partial_{Q}\Omega_{0}(C^{⋆},E){|}_{C,E}:[W_{I}\cdot Q]=0$, this results in

4.7 $$[\frac{\partial \Omega_{0}(C^{⋆},E)}{\partial C^{⋆}}\cdot \frac{\partial C^{⋆}}{\partial Q}\cdot Q^{t}]:W_{I}=\frac{1}{2}[S^{⋆}\cdot C^{⋆}-C^{⋆}\cdot S^{⋆}]:W_{I}=0\;\forall \;W_{I}=-W_{I}^{t},$$

cf. equation (4.2). Since $W_{I}$ is skewsymmetric, the coaxiality relation $S^{⋆}\cdot C^{⋆}=C^{⋆}\cdot S^{⋆}$ must hold, which is satisfied for E aligned with a principal direction $N_{i}^{⋆}$ of $C^{⋆}$, cf. equation (4.4). This also yields $\left[D^{⋆}⊗E\right]$ to be symmetric, cf. equation (4.3). Similar to equation (4.5), the second directional derivative

4.8 $$W_{I}:[\frac{\partial [S^{⋆}\cdot C^{⋆}-C^{⋆}\cdot S^{⋆}]}{\partial Q}\cdot Q^{t}]:W_{II}=W_{I}:{\text{T}}^{⋆}:W_{II}$$

can be considered, with

4.9 $$\begin{array}{rl} {\text{T}}^{⋆} & \;=\frac{1}{2}[I\bar{⊗}C^{⋆}]:{\text{E}}^{⋆}\cdot C^{⋆}-\frac{1}{2}[I\bar{⊗}C^{⋆}]:{\text{E}}^{⋆}:[C^{⋆}\bar{⊗}I]\\ & \;\;+\frac{1}{2}C^{⋆}\cdot {\text{E}}^{⋆}:[C^{⋆}\bar{⊗}I]-\frac{1}{2}C^{⋆}\cdot {\text{E}}^{⋆}\cdot C^{⋆}\\ & \;\;+S^{⋆}\bar{⊗}C^{⋆}-[S^{⋆}\cdot C^{⋆}]\bar{⊗}I+C^{⋆}\bar{⊗}S^{⋆}-I\bar{⊗}[S^{⋆}\cdot C^{⋆}] \end{array}$$

and ${\text{E}}^{⋆}=2\partial S^{⋆}/\partial C^{⋆}={\left[{\text{E}}^{⋆}\right]}^{T}$ as well as $W_{II}=-W_{II}^{t}$.

### (b) Decomposition of electromechanical coupling tensor

As previously mentioned, coaxiality of dielectric displacements and electric field result in specific symmetry properties of material tensors. In the following, the third-order tensor H=∂S/∂E, which is directly related to the electromechanical coupling properties, shall be specified. Based on the invariant representations in equations (3.1) and (3.2), together with $S=2\partial \Omega_{0}/\partial C$, one obtains

4.10 $$\begin{array}{rl} \frac{1}{2}\text{H} & \;=\sum_{i=1}^{3}\sum_{j=4}^{6}2i{\bar{\Omega }}_{0,ij}C^{i-1}⊗[C^{j-4}\cdot E]\\ & \;\;+\sum_{j=4}^{6}2[{\bar{\Omega }}_{0,5j}E⊗E+{\bar{\Omega }}_{0,6j}[C\cdot E⊗E+E⊗E\cdot C]]⊗[C^{j-4}\cdot E]\\ & \;\;+{\bar{\Omega }}_{0,5}[I\bar{⊗}E+E⊗I]+{\bar{\Omega }}_{0,6}[C\bar{⊗}E+E⊗C+C\cdot E⊗I+I\bar{⊗}[C\cdot E]] \end{array}$$

with ${\bar{\Omega }}_{0,ij}=\partial {\bar{\Omega }}_{0,i}/\partial I_{j}={\bar{\Omega }}_{0,ji}$, cf. equations (4.3) and (4.4).

The general symmetry property of H in the form V:H=0 for any skewsymmetric $V=-V^{t}$ can directly be concluded from either $S=S^{t}$ or equation (4.10). Further symmetry properties of H(C,E) are not directly obvious. Moreover, decompositions well established in computational mechanics, such as the spectral decomposition applied to second-order tensors, are not directly applicable to third-order tensors, respectively, H. In order to decompose H, which intrinsically represents electromechanical coupling properties, a harmonic decomposition is adopted so that particular symmetry properties may be reflected by the underlying harmonic tensors of H.

Following the approach proposed in [33], the third-order tensor H is decomposed into a fully symmetric part ${\text{H}}^{\mathrm{SYM}}$ and a remaining part ${\text{H}}^{\mathrm{SKW}}$, i.e. $\text{H}={\text{H}}^{\mathrm{SYM}}+{\text{H}}^{\mathrm{SKW}}$ with

4.11 $${\text{H}}^{\mathrm{SYM}}=\frac{1}{3}[\text{H}{+}^{T}\text{H}+{\text{H}}^{T}]\;\text{and}\;{\text{H}}^{\mathrm{SKW}}=\frac{1}{3}[2\text{H}{-}^{T}\text{H}-{\text{H}}^{T}].$$

The latter third-order tensor is related to the vector

4.12 $$g_{\text{H}}^{\mathrm{skw}}=\frac{1}{2}[2I\underline{⊗}I-I\bar{⊗}I-I⊗I]\cdot :\text{H}=I:\text{H}-\text{H}:I$$

by

4.13 $${\text{H}}^{\mathrm{SKW}}=\frac{1}{3}[2I⊗I-I\bar{⊗}I-I\underline{⊗}I]\cdot g_{\text{H}}^{\mathrm{skw}}.$$

In view of the fully symmetric part of H, a tensor ${\text{G}}_{\text{H}}$ is introduced satisfying the orthogonality condition ${\text{G}}_{\text{H}}\cdot :\text{H}=0$. The third-order tensor ${\text{G}}_{\text{H}}$ allows representation as

4.14 $${\text{G}}_{\text{H}}={\text{H}}^{\mathrm{SYM}}-\frac{1}{4}[I⊗I+I\bar{⊗}I+I\underline{⊗}I]\cdot g_{\text{H}}^{\mathrm{sym}}$$

whereby the vector $g_{\text{H}}^{\mathrm{sym}}$ is defined similarly to equation (4.12) but referred to the symmetric part, i.e.

4.15 $$g_{\text{H}}^{\mathrm{sym}}=\frac{1}{3}[I\underline{⊗}I+I\bar{⊗}I+I⊗I]\cdot :\text{H}=\frac{1}{3}[2I:\text{H}+\text{H}:I].$$

In summary, the third-order tensor H=∂S/∂E can be decomposed as

4.16 $$\text{H}={\text{G}}_{\text{H}}+\frac{1}{4}[I⊗I+I\bar{⊗}I+I\underline{⊗}I]\cdot g_{\text{H}}^{\mathrm{sym}}+\frac{1}{3}[2I⊗I-I\bar{⊗}I-I\underline{⊗}I]\cdot g_{\text{H}}^{\mathrm{skw}}$$

which, from a conceptual point of view, takes a similar interpretation as the well-established decomposition of a second-order tensor into a deviatoric symmetric part, a spherical contribution and a skewsymmetric term.

An alternative irreducible representation of ${\text{H}}^{\mathrm{SKW}}$ is established as

4.17 $${\text{H}}^{\mathrm{SKW}}=\frac{1}{3}[2I⊗m_{\text{H}}^{\mathrm{skw}}-I\bar{⊗}m_{\text{H}}^{\mathrm{skw}}-m_{\text{H}}^{\mathrm{skw}}⊗I]+\frac{1}{3}[M_{\text{H}}^{t}\cdot \varepsilon +{\left[\varepsilon \cdot M_{\text{H}}\right]}^{t}],$$

cf. [32], with ε denoting the third-order permutation tensor. Moreover, the relations

4.18 $$m_{\text{H}}^{\mathrm{skw}}=\frac{1}{2}g_{\text{H}}^{\mathrm{skw}}\;\text{and}\;M_{\text{H}}={\left[\text{H}:\varepsilon \right]}^{t}-\varepsilon \cdot m_{\text{H}}^{\mathrm{skw}}$$

have been used in equation (4.17). It is remarked that a second-order tensor similar to $M_{\text{H}}$ is not directly addressed in the representation summarized in equation (4.16) but $M_{\text{H}}$ explicitly senses skewsymmetric contributions of the form H:ε. Furthermore, a vectorial contribution related to the symmetric part of H is established as

4.19 $$m_{\text{H}}^{\mathrm{sym}}=\frac{1}{3}[I:\text{H}+2\text{H}:I]$$

together with the third-order contribution

4.20 $${\text{M}}_{\text{H}}={\text{H}}^{\mathrm{SYM}}-\frac{1}{5}[m_{\text{H}}^{\mathrm{sym}}⊗I+m_{\text{H}}^{\mathrm{sym}}\bar{⊗}I+I⊗m_{\text{H}}^{\mathrm{sym}}]$$

which, summarizing and by analogy with equation (4.16), yields a decomposition of the third-order tensor H=∂S/∂E in the form

4.21 $$\begin{array}{rl} \text{H} & \;={\text{M}}_{\text{H}}+\frac{1}{5}[m_{\text{H}}^{\mathrm{sym}}⊗I+m_{\text{H}}^{\mathrm{sym}}\bar{⊗}I+I⊗m_{\text{H}}^{\mathrm{sym}}]\\ & \;\;+\frac{1}{3}[2I⊗m_{\text{H}}^{\mathrm{skw}}-I\bar{⊗}m_{\text{H}}^{\mathrm{skw}}-m_{\text{H}}^{\mathrm{skw}}⊗I]+{\left[\varepsilon \cdot \varepsilon \cdot m_{\text{H}}^{\mathrm{skw}}\right]}^{t}]\\ & \;\;+\frac{1}{3}[M_{\text{H}}^{t}\cdot \varepsilon +{\left[\varepsilon \cdot M_{\text{H}}\right]}^{t}], \end{array}$$

cf. [32]. The vectors $m_{\text{H}}^{\mathrm{skw}}$ and tensors $m_{\text{H}}^{\mathrm{sym}}$, $M_{\text{H}}$ and ${\text{M}}_{\text{H}}$ are also denoted as deviators of H.

## 5. Examples

Two different homogeneous deformation states shall be elaborated in the following. The particular states studied are isochoric and consider different stretch ratios, whereby the orientation of the electric field is varied. To be specific, states with constant principal stretch directions and, in general, two identical principal stretches as well as three distinct principal stretches are investigated. As this work proceeds, the principal stretch directions $N_{1,2,3}$ are identified with a fixed orthonormal basis system $e_{1,2,3}$. The direction N of the electric field $E=\sum_{i=1}^{3}E_{i}N_{i}$ can be represented in spherical coordinates. One possible form is

5.1 $$E=EN\;\text{with}\;N=\cos(\theta_{1})\sin(\theta_{2})e_{1}+\sin(\theta_{1})\sin(\theta_{2})e_{2}+\cos(\theta_{2})e_{3}$$

so that $\theta_{2}\in [0,\pi ]$ denotes the angle between E and $e_{3}$ and $\theta_{1}\in [0,2\pi ]$ the angle between the projection of E onto the $e_{1,2}$-plane and $e_{1}$, respectively. Moreover, the magnitude of the electric field considered for the following examples is fixed to $E=5\times 10^{6}\;V\;m^{-1}$.

Emphasis shall be placed on the electromechanical coupling response as represented by the energy contribution $\Psi_{0}^{\mathrm{elm}}(C,E)$, cf. equation (3.9). Since electromechanical coupling is directly reflected by the third-order tensor H—in other words the sensitivity of stresses with respect to electric field and the sensitivity of dielectric displacements with respect to deformation, respectively—the related contribution ${\text{H}}^{\mathrm{elm}}$ shall be elaborated on. An electromechanical coupling energy contribution in the form of equation (3.15) is considered, namely

5.2 $${\tilde{\Psi }}_{0}^{\mathrm{elm}}=\sum_{s=1}^{M}\frac{\nu_{s}}{\beta_{s}}[\sum_{i=1}^{3}\tilde{f}E_{i}^{2}\lambda_{i}^{\beta_{s}}]=\sum_{s=1}^{M}\frac{\nu_{s}}{\beta_{s}}[\sum_{i=1}^{3}\tilde{f}[E⊗E]:\lambda_{i}^{\beta_{s}}[N_{i}⊗N_{i}]].$$

This yields the related Piola–Kirchhoff type stresses to take the representation

5.3 $$S^{\mathrm{elm}}=2\frac{\partial {\tilde{\Psi }}_{0}^{\mathrm{elm}}}{\partial C}=2\sum_{s=1}^{M}\frac{\nu_{s}}{\beta_{s}}[\sum_{i=1}^{3}\tilde{f}[E⊗E]:{\text{P}}_{i}+E_{i}^{2}\lambda_{i}^{\beta_{s}}\frac{\partial \tilde{f}}{\partial C}],$$

whereby ${\text{P}}_{i}=\partial [\lambda_{i}^{\beta_{s}}N_{i}⊗N_{i}]/\partial C$ is specified in remark 5.1. In this context, and by analogy with the derivation of $S^{\mathrm{elm}}$ based on equation (5.2), the related contribution to the dielectric displacements follows as

5.4 $$D^{\mathrm{elm}}=-\frac{\partial \Psi_{0}^{\mathrm{elm}}}{\partial E}=-2\sum_{s=1}^{M}\frac{\nu_{s}}{\beta_{s}}[\sum_{i=1}^{3}\tilde{f}E_{i}\lambda_{i}^{\beta_{s}}N_{i}].$$

It is noted that $\beta_{s}<0$, in combination with $\nu_{s}>0$, results in qualitatively similar stress response (of the stress contribution including ${\text{P}}_{i}$, see also equations (5.6) and (5.7)) compared to $\beta_{s}>0$ in combination with $\nu_{s}>0$, whereas the response in view of the dielectric displacements is qualitatively different for these cases as directly concluded from equation (5.4). Nevertheless, both approaches, choices are used and established in the literature, see e.g. [28,47] as well as equation (3.16).

With the specification of $S^{\mathrm{elm}}$ in equation (5.3) and $D^{\mathrm{elm}}$ in equation (5.4), respectively, the related third-order electromechanical coupling tensor can be derived as

5.5 $${\text{H}}^{\mathrm{elm}}=\frac{\partial S^{\mathrm{elm}}}{\partial E}=2\sum_{s=1}^{M}\frac{\nu_{s}}{\beta_{s}}[\sum_{i=1}^{3}\tilde{f}[{\text{P}}_{i}^{T}\cdot E+{\left[E\cdot {\text{P}}_{i}\right]}^{T}]+2E_{i}\lambda_{i}^{\beta_{s}}\frac{\partial \tilde{f}}{\partial C}⊗N_{i}].$$

For the subsequent examples, a basic particularization of the energy contribution in equation (5.2) is chosen which simplifies the representations of $S^{\mathrm{elm}}$, $D^{\mathrm{elm}}$ and ${\text{H}}^{\mathrm{elm}}$. To be specific, M=1 is chosen and the underlying parameters can be denoted as $\beta_{1}=\beta$ and $\nu_{1}=\nu$. By analogy with equation (3.16), $\tilde{f}=J$ is adopted so that $\partial \tilde{f}/\partial C=\frac{1}{2}JC^{-1}$. Moreover, the parameter ν is set to $\nu =\varepsilon_{0}[\varepsilon_{r}-1]$ with $\varepsilon_{r}=4.7$, cf. [19] and references cited therein, whereby the free space permittivity is $\varepsilon_{0}=8.854\times 10^{-12}\;N\;V^{-2}$. In order to simplify notation, ${\text{H}}^{\mathrm{elm}}$ shall be denoted as H in the following examples, even though the free space contribution ${\text{H}}^{\mathrm{fre}}=2\partial^{2}\Omega_{0}^{\mathrm{fre}}/\partial C\partial E$ is not included. Furthermore, norms of the deviators of H are considered which are defined as the square root of the scalar-valued contraction of the respective deviator with itself, to give an example $||\text{H}||={\left[\text{H}\cdot :\text{H}\right]}^{1/2}$.

**Remark 5.1.**

Different approaches and algorithms are established in the literature to calculate derivatives of the type presented in ${\text{P}}_{i}=\partial [\lambda_{i}^{\beta_{s}}N_{i}⊗N_{i}]/\partial C$; see e.g. [48–52]. Here, use is made of a formulation by analogy with the approach established in [48], see also [41]. Conceptually, the principal stretch based representation of the common elasticity tensor E=2∂S/∂C is used but with S replaced by $\lambda_{i}^{\beta_{s}}N_{i}⊗N_{i}$ (isotropic tensor function in C). In view of equation (5.3), this yields the derivative sought as

5.6 $$\begin{array}{rl} {\text{P}}_{i} & \;=\frac{\partial [\lambda_{i}^{\beta_{s}}N_{i}⊗N_{i}]}{\partial C}\\ & \;=\frac{1}{2}\sum_{j=1}^{3}\frac{1}{\lambda_{j}}\frac{\partial \lambda_{i}^{\beta_{s}}}{\partial \lambda_{j}}[N_{i}⊗N_{i}⊗N_{j}⊗N_{j}]\\ & \;\;+\frac{1}{2}\sum_{\begin{array}{c} j=1\\ j\neq i \end{array}}^{3}\frac{\lambda_{j}^{\beta_{s}}-\lambda_{i}^{\beta_{s}}}{\lambda_{j}^{2}-\lambda_{i}^{2}}[N_{i}⊗N_{j}⊗N_{i}⊗N_{j}+N_{i}⊗N_{j}⊗N_{j}⊗N_{i}] \end{array}$$

with $\partial \lambda_{i}^{\beta_{s}}/\partial \lambda_{j}=\beta_{s}\lambda_{i}^{\beta_{s}-1}\delta_{ij}$ so that

5.7 $$\frac{1}{2}\sum_{j=1}^{3}\frac{1}{\lambda_{j}}\frac{\partial \lambda_{i}^{\beta_{s}}}{\partial \lambda_{j}}[N_{i}⊗N_{i}⊗N_{j}⊗N_{j}]=\frac{1}{2}\beta_{s}\lambda_{i}^{\beta_{s}-2}N_{i}⊗N_{i}⊗N_{i}⊗N_{i}.$$

Moreover, in case of identical eigenvalues use of the established relation

5.8 $$\lim_{\lambda_{j}\to \lambda_{i}}\frac{\lambda_{j}^{\beta_{s}}-\lambda_{i}^{\beta_{s}}}{\lambda_{j}^{2}-\lambda_{i}^{2}}=\frac{1}{2}\beta_{s}\lambda_{j}^{\beta_{s}-2},$$

as based on de L’Hospital’s rule, is made. It is further remarked that the computation of the elasticity tensor E, as introduced in equation (2.6), includes the derivative $\partial {\text{P}}_{i}/\partial C$ when using the stress format in equation (5.3). It may turn out to be useful to derive E, if needed, by e.g. numerical or automatic differentiation approaches, see [53–55] and references cited therein.

### (a) Two identical principal stretches

The states of isochoric homogeneous deformations elaborated in the following possess constant principal stretch directions and, in general, two identical principal stretches. This results in the deformation gradient

5.9 $$F=\frac{1}{\sqrt{\lambda_{3}}}[e_{1}⊗e_{1}+e_{2}⊗e_{2}]+\lambda_{3}e_{3}⊗e_{3}$$

so that $\lambda_{1}=\lambda_{2}=1/\sqrt{\lambda_{3}}$ and det(F)=1, $F=F^{\mathrm{iso}}$, respectively.

Dependencies on $\lambda_{3}$ and on the direction of the electric field as represented by $\theta_{1}$ and $\theta_{2}$, cf. equation (5.1), are investigated in the following. Since the stretches in the $e_{1,2}$-plane are identical, angle $\theta_{1}=0$ is not varied but $\theta_{2}$ changed within the interval [0,π/2]. In other words, the electric field considered lies in the $e_{1,3}$-plane and remains perpendicular with respect to $e_{2}$. Moreover, different values of the parameter β, cf. equation (5.2), are elaborated on. In particular, positive and negative values can be chosen for β which motivates the choice β=±3/2 for the subsequent examples.

**Figure 1.**
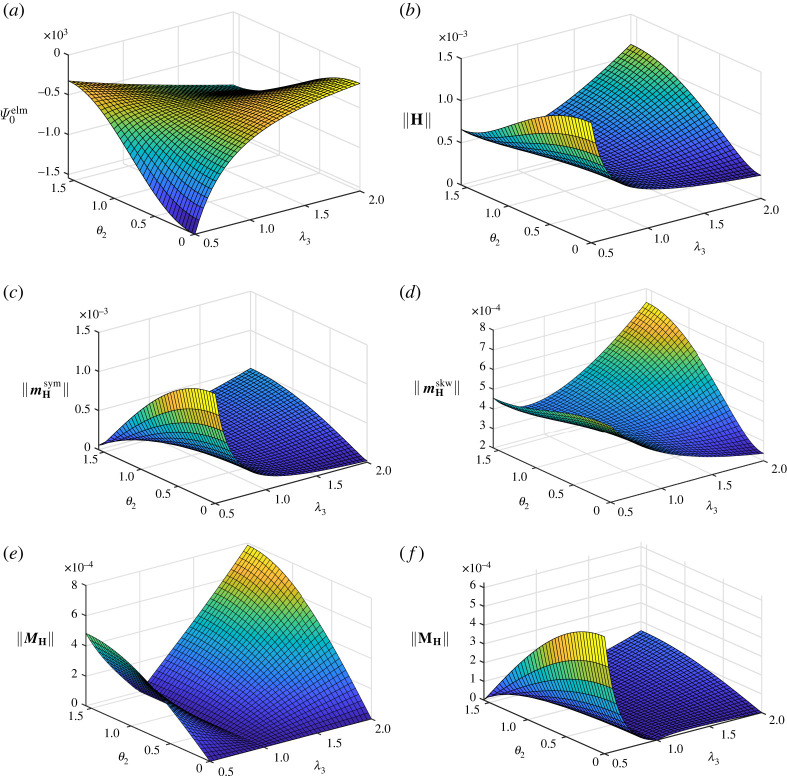
Homogeneous isochoric deformation with $\lambda_{1}=\lambda_{2}=1/\sqrt{\lambda_{3}}$: illustrations of the electromechanical energy contribution $\Psi_{0}^{\text{elm}}$, the norm of the electromechanical coupling tensor H and its deviators for material parameter β=−3/2. (*a*) Electromechanical energy contribution $\Psi_{0}^{\text{elm}}$ in ${N\;m}^{-2}$. (*b*) Norm of the electromechanical coupling tensor H in $N\;V^{-1}\;m^{-1}$. (*c*) Norm of the first-order symmetric deviator $m_{\text{H}}^{\mathrm{sym}}$ of H in $N\;V^{-1}\;m^{-1}$. (*d*) Norm of the first-order skewsymmetric deviator $m_{\text{H}}^{\mathrm{skw}}$ of H in $N\;V^{-1}\;m^{-1}$. (*e*) Norm of the second-order deviator $M_{\text{H}}$ of H in $N\;V^{-1}\;m^{-1}$. (*f*) Norm of the third-order deviator ${\text{M}}_{\text{H}}$ of H in $N\;V^{-1}\;m^{-1}$. (Online version in colour.)

Emphasis is placed on the electromechanical coupling response under changing orientations of the electric field and related results for β=−3/2 are shown in figure 1. The electromechanical coupling contribution to the augmented energy is displayed in figure 1*a* in dependence of $\lambda_{3}$ and $\theta_{2}$, where the influence of the negative value chosen for β is clearly visible, i.e. $\Psi_{0}^{\mathrm{elm}}\leq 0$. The electric field E=EN is aligned with a principal stretch direction for $\theta_{2}=0$, so that $N=e_{3}$, and for $\theta_{2}=\pi /2$, where $N=e_{1}$. It is seen that $\Psi_{0}^{\mathrm{elm}}\leq 0$ takes extremal values for these orientations of E for fixed values of $\lambda_{3}$, whereby maximum (minimum) values are obtained if these particular directions N are loaded under tension (compression). The norm of H and the norms of the related deviators are illustrated in figure 1*b*–*f*, whereby the norm of H in figure 1*b* highlights loading dependent electromechanical properties in general. As expected, the graph of ||H|| with respect to $\lambda_{3}$ and $\theta_{2}$ shows similar characteristics to a related graph of $|\Psi_{0}^{\mathrm{elm}}|$. In view of the norms of the deviators, special emphasis is placed on the norm of the second-order deviator $M_{\text{H}}$ in figure 1*e*. It is observed that $||M_{\text{H}}||=0$ is independent of $\lambda_{3}$ for $\theta_{2}=0$. This orientation of the electric field yields E not only aligned to a principal stretch direction, and by that corresponding to an extremal state of energy, but perpendicular to the ($e_{1,2}$–) plane spanned by the principal directions related to the coinciding principal stretches $\lambda_{1}=\lambda_{2}$. Such states are often used for electromechanical devices undergoing large changes of deformation. In view of the design of these devices, a vanishing second-order deviator $M_{\text{H}}$ can be used as an indicator of optimal operation states. As a special case, the mechanically initial undeformed state is obtained for $\lambda_{1}=\lambda_{2}=\lambda_{3}=1$, for which the norms of the second-order deviator, $||M_{\text{H}}||$, and of the third-order deviator, $||{\text{M}}_{\text{H}}||$, both vanish identically independent of $\theta_{2}$, cf. figure 1*e*,*f*.

**Figure 2.**
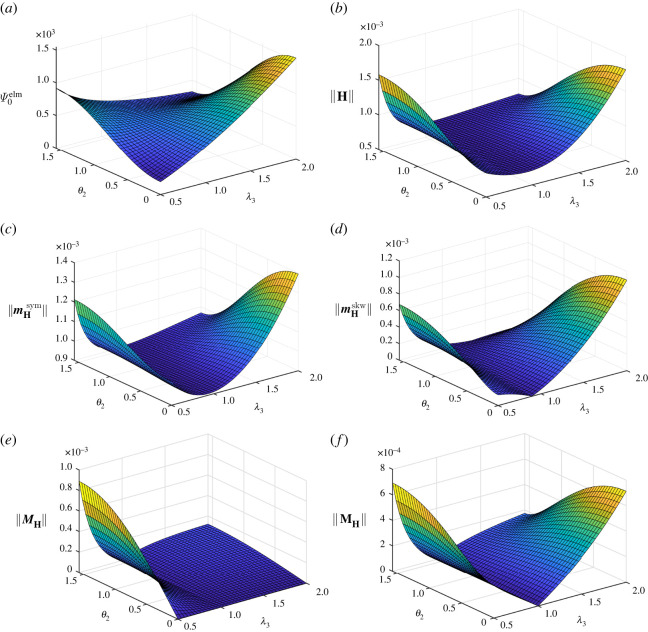
Homogeneous isochoric deformation with $\lambda_{1}=\lambda_{2}=1/\sqrt{\lambda_{3}}$: illustrations of the electromechanical energy contribution $\Psi_{0}^{\text{elm}}$, the norm of the electromechanical coupling tensor H and its deviators for material parameter β=3/2. (*a*) Electromechanical energy contribution $\Psi_{0}^{\text{elm}}$ in $N\;m^{-2}$. (*b*) Norm of the electromechanical coupling tensor H in $N\;V^{-1}\;m^{-1}$. (*c*) Norm of the first-order symmetric deviator $m_{\text{H}}^{\mathrm{sym}}$ of H in $N\;V^{-1}\;m^{-1}$. (*d*) Norm of the first-order skewysymmetric deviator $m_{\text{H}}^{\mathrm{skw}}$ of H in $N\;V^{-1}\;m^{-1}$. (*e*) Norm of the second-order deviator $M_{\text{H}}$ of H in $N\;V^{-1}\;m^{-1}$. (*f*) Norm of the third-order deviator ${\text{M}}_{\text{H}}$ of H in $N\;V^{-1}\;m^{-1}$. (Online version in colour.)

By analogy with figure 1, related results for β=3/2 are shown in figure 2. The electromechanical coupling contribution to the augmented energy displayed in figure 2*a* shows a similar characteristic to $\Psi_{0}^{\mathrm{elm}}$ in figure 1*a*, but $\Psi_{0}^{\mathrm{elm}}\geq 0$ for β=3/2 in figure 2*a*. It is interesting to note that the graphs of ||H|| and $||m_{\text{H}}^{\mathrm{sym}}||$ in figure 1*b*,*c*, for β=−3/2, as well as in figure 1*b*,*c*, for β=3/2, respectively, show similar characteristics. By analogy with the results obtained for β=−3/2, β=3/2 also yields the norm $||M_{\text{H}}||=0$ for $\theta_{2}=0$, i.e. independent of $\lambda_{3}$, cf. figure 2*e*. Moreover, $||M_{\text{H}}||$ and $||{\text{M}}_{\text{H}}||$ both vanish identically for $\lambda_{1}=\lambda_{2}=\lambda_{3}=1$, independent of $\theta_{2}$, cf. figure 2*e*,*f*.

### (b) Three distinct principal stretches

By analogy with the isochoric homogeneous states of deformation considered in §5a, constant principal stretch directions but, in general, three distinct principal stretches are accounted for. In consequence, the underlying deformation gradient reads

5.10 $$F=\frac{1}{\lambda_{2}\lambda_{3}}e_{1}⊗e_{1}+\lambda_{2}e_{2}⊗e_{2}+\lambda_{3}e_{3}⊗e_{3}$$

so that det(F)=1 and $F=F^{\mathrm{iso}}$, respectively. For the following examples, a fixed compressive stretch $\lambda_{3}=0.8$ is considered and $\lambda_{2}$ is varied so that $\lambda_{1}=1/[0.8\lambda_{2}]$.

Similar to the results shown in figures 1 and 2, dependencies on $\lambda_{2}$ and on the direction of the electric field as represented by $\theta_{1}$ and $\theta_{2}$, cf. equation (5.1), are investigated in the following. Since changes in the stretches $\lambda_{1}$ and $\lambda_{2}$ as referred to the $e_{1,2}$-plane are considered in the subsequent elaborations, a fixed projection direction of E onto the $e_{1,2}$-plane shall be considered. To be specific, $\theta_{1}=\pi /2$ is chosen while $\theta_{2}$ is changed within the interval [0,π/2]. In other words, the electric field considered lies in the $e_{2,3}$-plane and remains perpendicular with respect to $e_{1}$. By analogy with §5a, different values of the parameter β, cf. equation (5.2), are studied, i.e. β=±3/2 is adopted.

**Figure 3.**
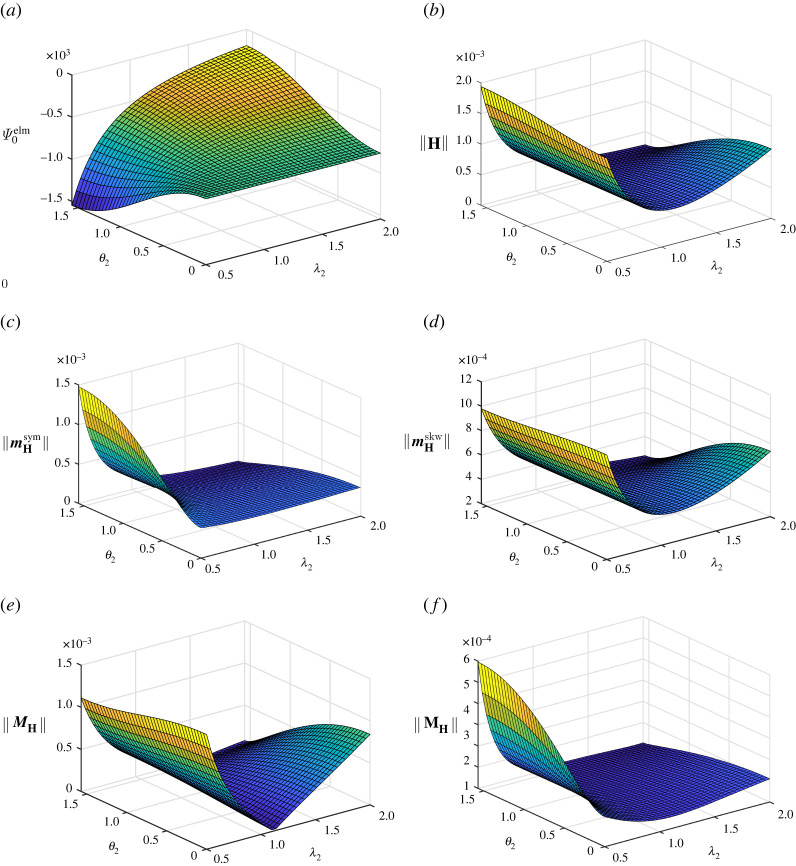
Homogeneous isochoric deformation with $\lambda_{1}=1/[\lambda_{2}\lambda_{3}]$: illustrations of the electromechanical energy contribution $\Psi_{0}^{\text{elm}}$, the norm of the electromechanical coupling tensor H and its deviators for material parameter β=−3/2. (*a*) Electromechanical energy contribution $\Psi_{0}^{\text{elm}}$ in $N\;m^{-2}$. (*b*) Norm of the electromechanical coupling tensor H in $N\;V^{-1}\;m^{-1}$. (*c*) Norm of the first-order symmetric deviator $m_{\text{H}}^{\mathrm{sym}}$ of H in $N\;V^{-1}\;m^{-1}$. (*d*) Norm of the first-order skewsymmetric deviator $m_{\text{H}}^{\mathrm{skw}}$ of H in $N\;V^{-1}\;m^{-1}$. (*e*) Norm of the second-order deviator $M_{\text{H}}$ of H in $N\;V^{-1}\;m^{-1}$. (*f*) Norm of the third-order deviator ${\text{M}}_{\text{H}}$ of H in $N\;V^{-1}\;m^{-1}$. (Online version in colour.)

Figure 3 shows results obtained for β=−3/2. It is clearly seen in figure 3*a* that this choice yields $\Psi_{0}^{\mathrm{elm}}\leq 0$. Moreover, the electromechanical coupling contribution to the augmented energy turns out, as expected, to be independent of $\lambda_{2}$ in case $\theta_{2}=0$, i.e. $N=e_{3}$. Extremal values of $\Psi_{0}^{\mathrm{elm}}\leq 0$ at fixed $\lambda_{2}$ are obtained at $\theta_{2}=\pi /2$, in other words when E is aligned with $e_{2}$, which is a principal stretch direction. In such cases, i.e. $\theta_{2}=\pi /2$, the value of $\Psi_{0}^{\mathrm{elm}}$ increases with $\lambda_{2}$. By analogy with the results in figure 1, the graph of ||H|| shown in figure 3*b* possesses similar characteristics to a related graph of $|\Psi_{0}^{\mathrm{elm}}|$ referred to in figure 3*a*. Concerning the norms of the deviators displayed in figure 3*c*–*f*, special emphasis is placed on the norm of the second-order deviator $M_{\text{H}}$ in figure 3*e*. The stretch value $\lambda_{2}=1/\sqrt{\lambda_{3}}\approx 1.118$ results in $\lambda_{1}=\lambda_{2}$. In that case, $||M_{\text{H}}||=0$ for $\theta_{2}=0$, in other words a state where E is perpendicular to a plane related to identical principal stretches. This is, as mentioned before, often the case for finite deformation electromechanical devices. The same property, i.e. $||M_{\text{H}}||=0$, is observed for $\lambda_{2}=1/0.8^{2}=1.5625$, so that $\lambda_{1}=\lambda_{3}$ and $\theta_{2}=\pi /2$ yield the electric field E aligned with $e_{2}$. Furthermore, it is remarked that $||M_{\text{H}}||\neq 0$ for $\lambda_{2}=\lambda_{3}=0.8$, since $\theta_{1}=\pi /2$ so that E cannot be aligned with $e_{1}$, i.e. a direction perpendicular to the plane spanned by the principal directions related to $\lambda_{2}$ and $\lambda_{3}$.

**Figure 4.**
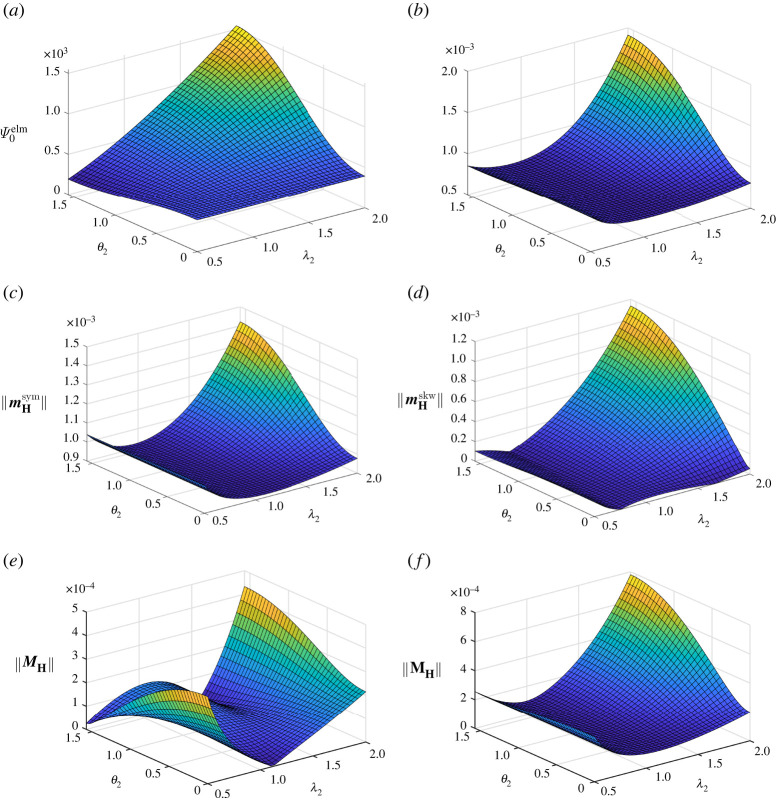
Homogeneous isochoric deformation with $\lambda_{1}=1/[\lambda_{2}\lambda_{3}]$: illustrations of the electromechanical energy contribution $\Psi_{0}^{\text{elm}}$, the norm of the electromechanical coupling tensor H and its deviators for material parameter β=3/2. (*a*) Electromechanical energy contribution $\Psi_{0}^{\text{elm}}$ in $N\;m^{-2}$. (*b*) Norm of the electromechanical coupling tensor H in $N\;V^{-1}\;m^{-1}$. (*c*) Norm of the first-order symmetric deviator $m_{\text{H}}^{\mathrm{sym}}$ of H in $N\;V^{-1}\;m^{-1}$. (*d*) Norm of the first-order skewsymmetric deviator $m_{\text{H}}^{\mathrm{skw}}$ of H in $N\;V^{-1}\;m^{-1}$. (*e*) Norm of the second-order deviator $M_{\text{H}}$ of H in $N\;V^{-1}\;m^{-1}$. (*f*) Norm of the third-order deviator ${\text{M}}_{\text{H}}$ of H in $N\;V^{-1}\;m^{-1}$. (Online version in colour.)

By analogy with figure 3, as well as with figure 2, related results for β=3/2 are displayed in figure 4. The graph of the energy contribution $\Psi_{0}^{\mathrm{elm}}$ shown in figure 4*a* possesses, in general, similar characteristics to the one displayed for β=−3/2 in figure 3*a*, but $\Psi_{0}^{\mathrm{elm}}\geq 0$ for β=3/2 in figure 4*a*. The graphs of the norm of H and of the norms of the deviators of H are highlighted in figure 4*b*–*f* with respect to $\theta_{2}$ and $\lambda_{2}$. As expected, i.e. independent of the particular choice of β, the norm of the second-order deviator $||M_{\text{H}}||$ vanishes, in cases where the orientation of the electric field E is perpendicular to a plane spanned by principal stretch direction related to identical principal stretch values. As discussed for the previous example with β=−3/2 highlighted in figure 3, this is the case for $\lambda_{2}=1/\sqrt{\lambda_{3}}\approx 1.118$ and $\theta_{2}=0$, as well as for $\lambda_{2}=1.5625$, so that $\lambda_{1}=\lambda_{3}=0.8$, and $\theta_{2}=\pi /2$, cf. figure 4*e*.

## 6. Summary

The main goal of this paper is to study extremal (local) states of energy for finite deformation electroelasticity together with electromechanical coupling properties. The general framework adopted in this work for the modelling of electroelasticity makes use of an augmented free energy contribution. This energy is additionally split into a purely elastic contribution, an electromechanical coupling term and a free space part. The general representation of these energy contributions in terms of invariants related to deformation measures, electric field and dielectric displacements, respectively, is well-established—although the particularization of the energy function, as a basis for electromechanical constitutive models to precisely predict experimental data, remains challenging. This holds in particular for the electromechanical coupling contributions, whereas in view of model fitting and parameter identification comprehensive constitutive frameworks, such as the celebrated Ogden model, are established. In this context, an electromechanical coupling function of Ogden model type has been proposed in this work, which combines quadratic contributions in the electric field with powers of stretches, both referred to as the principal stretch directions. This particular format may also turn out to be useful in view of parameter identification, i.e. matching predictive simulations with experimental data. As a side aspect, the formulation includes transversely isotropic elasticity as a special case. In order to analyse electromechanical coupling properties, the third-order electromechanical coupling tensor, i.e. the derivative of stresses with respect to electric field, has been studied. As a basis, a tensor decomposition of this third-order tensor is applied, which results in four deviators, two of which are vectors, one is a second- and one a third-order tensor. The investigation of two different types of homogeneous states of deformation with changing orientation of electric field and two different (sets of) material parameters for the electromechanical coupling contribution showed that for some of the particular deformations studied the norm of a few deviators, e.g. of first and third order, exhibit similar characteristics to the norm of the third-order electromechanical coupling tensor itself. Of special interest is that the second-order deviator is identically zero for particular states of deformation. These particular states correspond to (local) extremal states of energy where, in addition, two principal stretches are identical and the electric field is perpendicular to the plane spanned by the principal direction related to these identical principal stretches. Such states are often used for electromechanical devices undergoing large deformations. In this regard, the analysis may contribute to computationally predicting and illustrating such states that are attractive from a design and performance perspective, and optimization viewpoint, respectively, of electroelastic devices. To give an example, the design of energy harvesting devices, see e.g. [47,56,57] among other references, may benefit from operating in such states and a robust design so that geometrical imperfections do not significantly influence such states. The interpretation and visualization of the deviators of the third-order electromechanical coupling tensor remains challenging, however, and needs further investigation in future. Finally, the approach proposed may also be useful and of interest for other fields of applications, e.g. in the area of magnetoelasticity.

## Data Availability

This article has no additional data.
